# Multi-Faceted Effects of ST6Gal1 Expression on Precursor B-Lineage Acute Lymphoblastic Leukemia

**DOI:** 10.3389/fonc.2022.828041

**Published:** 2022-03-16

**Authors:** Mingfeng Zhang, Tong Qi, Lu Yang, Daniel Kolarich, Nora Heisterkamp

**Affiliations:** ^1^ Department of Systems Biology, Beckman Research Institute City of Hope, Duarte, CA, United States; ^2^ Institute for Glycomics, Griffith University, Gold Coast, QLD, Australia; ^3^ Australian Research Council (ARC) Centre of Excellence for Nanoscale BioPhotonics, Griffith University, Gold Coast, QLD, Australia

**Keywords:** sialyltransferase, BCP-ALL, drug resistance, vincristine, microenvironment, N-linked glycan, α2-6 sialic acid

## Abstract

Normal early human B-cell development from lymphoid progenitors in the bone marrow depends on instructions from elements in that microenvironment that include stromal cells and factors secreted by these cells including the extracellular matrix. Glycosylation is thought to play a key role in such interactions. The sialyltransferase ST6Gal1, with high expression in specific hematopoietic cell types, is the only enzyme thought to catalyze the terminal addition of sialic acids in an α2-6-linkage to galactose on N-glycans in such cells. Expression of ST6Gal1 increases as B cells undergo normal B-lineage differentiation. B-cell precursor acute lymphoblastic leukemias (BCP-ALLs) with differentiation arrest at various stages of early B-cell development have widely different expression levels of *ST6GAL1* at diagnosis, with high *ST6Gal1* in some but not in other relapses. We analyzed the consequences of increasing ST6Gal1 expression in a diagnosis sample using lentiviral transduction. NSG mice transplanted with these BCP-ALL cells were monitored for survival. Compared to mice transplanted with leukemia cells expressing original ST6Gal1 levels, increased ST6Gal1 expression was associated with significantly reduced survival. A cohort of mice was also treated for 7 weeks with vincristine chemotherapy to induce remission and then allowed to relapse. Upon vincristine discontinuation, relapse was detected in both groups, but mice transplanted with ST6Gal1 overexpressing BCP-ALL cells had an increased leukemia burden and shorter survival than controls. The BCP-ALL cells with higher ST6Gal1 were more resistant to long-term vincristine treatment in an *ex vivo* tissue co-culture model with OP9 bone marrow stromal cells. Gene expression analysis using RNA-seq showed a surprisingly large number of genes with significantly differential expression, of which approximately 60% increased mRNAs, in the ST6Gal1 overexpressing BCP-ALL cells. Pathways significantly downregulated included those involved in immune cell migration. However, ST6Gal1 knockdown cells also showed increased insensitivity to chemotherapy. Our combined results point to a context-dependent effect of ST6Gal1 expression on BCP-ALL cells, which is discussed within the framework of its activity as an enzyme with many N-linked glycoprotein substrates.

## Introduction

B-cell precursor acute lymphoblastic leukemia (BCP-ALL) is a collective name for leukemias with differentiation arrest at various stages of early B-cell development. Owing to extensive molecular analysis including gene expression and DNA sequencing, it is possible to distinguish up to 23 different subcategories of BCP-ALL (1). However, very little is known regarding the glycome of such leukemias. Glycosylation is a dynamic and highly abundant protein post-translational modification in which glycans are attached to proteins or lipids by controlled biosynthetic pathways. Glycoproteins and glycolipids are major constituents of the cell surface glycocalyx, the major zone involved in all intercellular interactions. Glycosylation is applied by the consecutive and controlled action of numerous glycosyltransferases located in the endoplasmic reticulum and Golgi stack. Main sites of glycan attachment in glycoproteins are at serine/threonine [O-glycans] or asparagine [N-glycans] residues (2).

Sialyltransferases (ST), which attach sialic acids [Sia] as the final monosaccharide to such glycan structures, are of particular significance due to the unique biochemical properties of Sia. Sias are attached by specific sialyltransferases ST3Gal, ST6Gal/ST6GalNAc, and ST8Sia to glycoproteins in α2-3, α2-6, or α2-8 glycosidic linkages, respectively. The exact linkage has biological significance: carbohydrate-binding proteins [lectins] have evolved to recognize such specific linkages, forming the biological basis of, for example, species-restricted influenza infection (3) and specific binding by Siglecs such as the B-cell inhibitory CD22 (4). As a consequence, Sias play a crucial role in numerous signaling pathways including but not limited to those regulating Siglec signaling in innate and adaptive immunity (5).

There are only two human ST6Gal enzymes known to attach Sia onto N-glycans in an α2-6 linkage. ST6Gal2 is expressed mainly in neuronal tissues and in the thyroid gland (6), whereas ST6Gal1 is ubiquitously expressed, with highest levels in the liver and hematopoietic tissues (7). ST6Gal1 is the most intensively studied sialyltransferase in cancer. Increased ST6Gal1 expression was reported in pancreatic, prostate, breast, and ovarian cancer, and was implicated as contributing to tumor growth, metastasis, and signal transduction pathways relevant to tumorigenesis (8–15). Nonetheless, the possible active contribution of this enzyme to carcinomas is also controversial (16).

ST6Gal1 is known to sialylate many well-known cell-surface glycoproteins as demonstrated by exogenous enzymatic assays on different cell lines [HEL, HeLa and mouse lung (17–19)]. In human HEL cells, which were established from a patient with Hodgkin’s disease, the 100 different substrates identified included for example CD44, numerous integrins, ICAMs, IGF1R, NOTCH1/2, and PTPRC/CD45. Since many of these glycoproteins contribute to cancer, sialylation is viewed as important from a potential diagnostic, therapeutic, and mechanistic viewpoint (20–22). ST6Gal1 also modifies the activity of the cell surface adhesion receptor PECAM1 and the store-operated calcium channel Oria1 (23, 24). Thus, increased expression of ST6Gal1 could contribute to tumorigenesis by Sia modification of many different cell surface glycoproteins, regulating cell–cell interactions and differential intracellular signaling through this route. However, the information regarding which glycoproteins are substrates of specific STs is limited because it requires analytical ability to discriminate Sia linkage in a protein-specific context.

Recently, we compared the glycome of primary B-lineage MLL-r leukemia, a subgroup of BCP-ALL, with that of normal bone marrow control CD19+CD10+ pre-B cells. Interestingly, we found increased levels of sialylated N-glycans, including α2-6 sialic acid-linked glycoconjugates, in the leukemia samples despite a downregulation of *ST6GAL1* on a transcript level (25). We considered that such higher levels of N-linked α2-6 Sia in primary BCP-ALL cells could have functional consequences, but a possible contribution of ST6Gal1 to BCP-ALL has not been examined. To test this, we here overexpressed *ST6GAL1* in a diagnosis BCP-ALL and found that in this BCP-ALL, high levels of ST6Gal1 associate with increased malignancy and large effects on the transcriptome of the cells.

## Results

### BCP-ALL Cells Have Extensive α2,6 Sialylation With High but Varying Levels of *ST6GAL1* mRNA Expression

**Figure 1:**
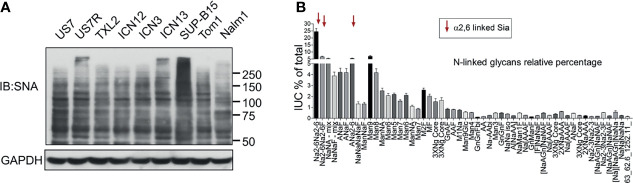
BCP-ALL cells contain high levels of α2-6 sialylation. **(A)** Western blot of different BCP-ALL cell lines probed with SNA lectin to specifically detect α2-6-linked sialic acids on glycoproteins. GAPDH, loading control. Location of molecular weight standards to the right. **(B)** Analysis of N-glycans in RS4;11 cells as previously described (25). Combined results of 15 individual RS4;11 cell samples. Overall, more than 65% of all identified N-glycans were found to be sialylated with 7.4% in α2-3, 14.1% in α2-3/6, and 45.1% in α2-6 attachment.

α2-6 sialylation can be detected by the lectin SNA. We used it to examine this specific Sia linkage in glycoproteins of a number of different PDX-derived as well as established, suspension-propagated BCP-ALL cell lines. As shown in **Figure 1A**, when used as a Western blot probe, SNA detects many glycoproteins, and/or different glycoforms of the same protein in BCP-ALL cell lines indicating that ST6Gal1 can sialylate numerous substrates in this type of leukemia. FACS analysis using SNA confirmed that there was, overall, very high representation of α2-6-linked Sia on the cell surface of such cells [for example, see **Supplementary Figure 1B**, negative controls US7, LAX57, and LAX56]. We also analyzed the relative abundance of α2-6-linked Sia using analytical glycan methods on RS4;11 as an example of a widely studied BCP-ALL suspension cell line. We found that, overall, 65% of N-linked glycans were capped by sialylation. Structures carrying Sia in α2-6-linkage were the single most abundant (>45%) modification, with fewer α2-3 Sias-containing glycans (**Figure 1B**).

**Figure 2:**
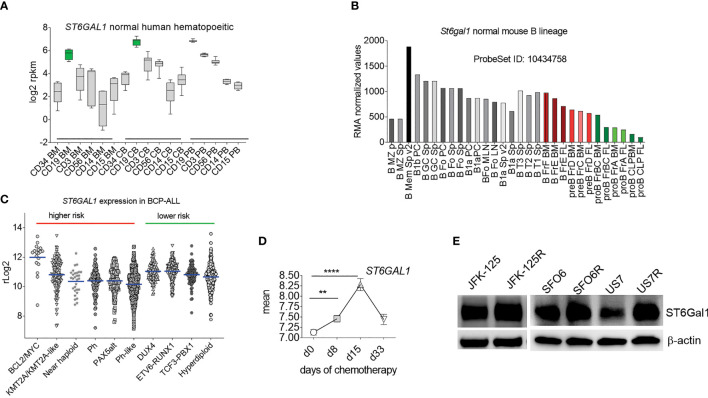
*ST6GAL1* is highly expressed in normal and abnormal B-lineage cells. **(A)** RNA-seq-based expression levels of *ST6GAL1* in normal human hematopoietic cells (1). BM, bone marrow; CB, cord blood; PB, peripheral blood. Cells were sorted for the indicated major lineage markers [CD19: B- cells; CD3: T-cells; CD56: NK cells; CD14: myeloid/macrophage; CD15: myeloid]. Green: CD19 BM *n* = 4; CD19 CB *n* = 10; CD19 PB *n* = 7. **(B)** Normalized RMA values of *ST6gal1* expression in murine hematopoietic cell types [GSE15907]. FL, fetal liver. Sp, spleen; LN, lymph node. Colored bars: B-cell developmental stages located in the bone marrow. **(C)** Scatter dot plot of rLog2 expression of *ST6GAL1* across selected subcategories of human BCP-ALL samples as indicated. Blue lines, mean values. **(D)** *ST6GAL1* RNA expression in pediatric ALL during chemotherapy treatment. Each symbol represents the mean ± SEM at an individual time point. Mean log-transformed normalized GEP values in 220 pediatric *de novo* ALL at diagnosis, day 8, day 15, and day 33 of remission–induction therapy [GSE67684]. **p < 0.01; ****p < 0.0001. Source of expression data, see **Supplementary Table 4**. **(E)** Western blot of the indicated diagnosis and relapsed (R) samples from the same patient. β-actin, loading control.

These results are in agreement with our glycan analysis of primary BCP-ALL patient samples (25). We conclude that N-glycan-linked α2-6 sialylation is a very common glycan-capping modification in RS4;11 and primary BCP-ALL cells. Because ST6Gal1 is thought to be the only glycosyltransferase responsible for this modification, we examined its expression in hematopoietic cell types. As shown in **Figure 2A**, normal human hematopoietic cells differ in *ST6GAL1* expression, with relatively lower levels in myeloid, and highest levels in CD19+ B-lineage cells. CD34+ bone marrow progenitor cells also have relatively low *ST6GAL1* mRNA consistent with reports of low *St6gal1* expression in HSPC in mice (26). Within normal B-lineage development in the mouse (**Figure 2B**) and human (**Supplementary Figure 2**), progression from pro-B to more mature B cells correlates with increased *St6gal1* mRNA levels. However, in diagnosis human BCP-ALL samples, expression of *ST6GAL1* showed a more than 300-fold variability between the highest and lowest levels with no correlation (**Figure 2C**) between expression levels and mutation-associated risk category (27–29). In a sample set of pediatric BCP-ALL treated with induction chemotherapy over 33 days, a significant increase in expression occurred on day 15 of chemotherapy (**Figure 2D**), suggesting that *ST6GAL1* expression may additionally be regulated by inflammation as reported (30–32), which could be caused by drug treatment and/or ensuing cell death. Using Western blotting, we also measured ST6Gal1 (**Figure 2E**) in three sets of BCP-ALLs for which we had matched relapse/diagnosis samples (**Supplementary Table 1**) and that grew in tissue co-culture. Overall, these analyses showed that *ST6GAL1* is ubiquitously expressed, but at varying levels in B-lineage cells.

### Increased ST6Gal1 Expression in US7 BCP-ALL Cells Promotes More Rapid Leukemia Cell Expansion in Mice

**Figure 3:**
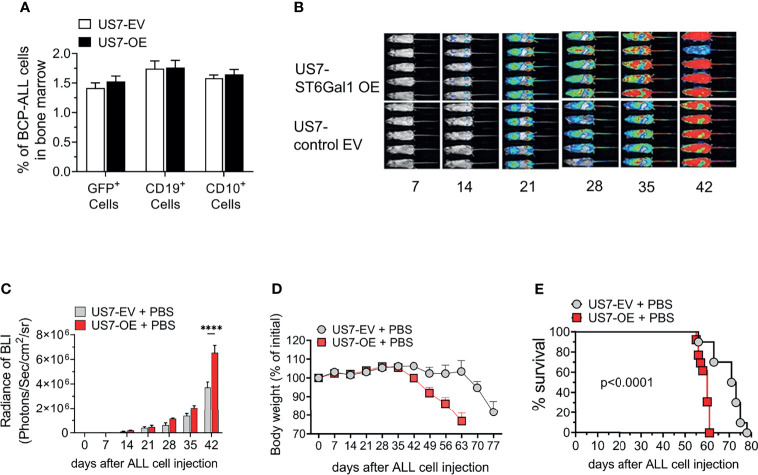
NSG mice transplanted with US7 ST6Gal1 overexpressing ALL cells have decreased survival. **(A)** Homing of US7 ST6Gal1 OE or US7 EV leukemia cells to bone marrow of mice 16 h after i.v. injection. *n* = 3/group **(B, C)** Bioluminescent imaging of female cohort (*n* = 5/group) over time. ****p < 0.001, adjusted *p*-values, Šidák’s multiple comparison test. Cells for transplant were transduced with a LV encoding luciferase. **(D)** Body weight changes and **(E)** survival of combined male and female cohorts [*n* = 10–13 total mice per group]. Kaplan–Meier survival curve comparing US7 control EV with US7 ST6Gal1 OE-transplanted mice. ****p < 0.0001, Log-rank test.

To investigate whether or not increased ST6Gal1 expression can contribute to a more malignant phenotype in cells that initially have relatively lower expression, we transduced US7 BCP-ALL cells with a vector encoding human ST6Gal1 (**Supplementary Figure 1A**) or with the empty vector, then flow-sorted cells to obtain a homogenous population. When we compared the ability of these cells to home to the bone marrow after i.v. injection in NSG mice, no significant differences were measured (**Figure 3A**). We next transplanted the cells into NSG mice to monitor leukemia development. Based on bioluminescence (**Figures 3B, C**), mice transplanted with high ST6Gal1-expressing BCP-ALL cells showed a more rapid leukemia expansion compared to the controls and more rapid body weight loss (**Figure 3D**). Also, compared to mice transplanted with leukemia cells expressing original ST6Gal1 levels, increased ST6Gal1 expression was associated with significantly reduced survival (**Figure 3E**).

**Figure 4:**
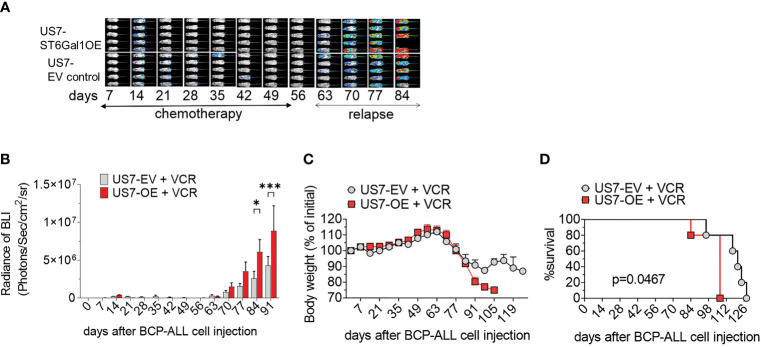
Vincristine-induced remission and relapse of NSG mice transplanted with US7 ST6Gal1 OE or control EV cells. Female mice were transplanted with 2 × 10^6^ cells on d0. Vincristine i.p. treatment was started on day 14 after transplant and was administered once per week at 0.5 mg/kg. **(A)** Bioluminescent images (BLI) of mice and **(B)** BLI quantification at weekly intervals of the two cohorts. *n* = 5 female mice/group. Two-way ANOVA, adjusted *p*-values, Šidák’s multiple comparison test. *p < 0.05; ***p < 0.001. **(C)** Body weight loss **(D)** Overall survival. **p = 0.0047, Log-rank test.

To compare the *in vivo* response to chemotherapy of these leukemia cells, we transplanted them into mice and allowed the leukemia cells to proliferate for 14 days before starting vincristine treatment. In the first weeks of treatment, based on bioluminescent imaging, chemotherapy was able to effectively control the expansion of the leukemia cells (**Figures 4A, B**, days 7–56). Treatment was discontinued after week 8, and relapse in both groups became evident about 14 days later. Based on bioluminescent imaging (**Figure 4A**, relapse; **Figure 4B**) and body weight loss (**Figure 4C**), US7 cells with increased expression of ST6Gal1 expanded and caused terminal leukemia more rapidly than the controls (**Figure 4D**). Thus, *in vivo*, increased ST6Gal1 expression allowed BCP-ALL cells to expand more rapidly than BCP-ALL cells with lower levels of ST6Gal1.

### Contribution of ST6Gal1 Overexpression to Chemotherapy Resistance

**Figure 5:**
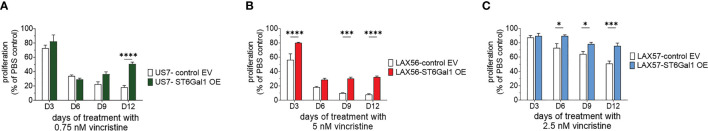
BCP-ALLs with ST6Gal1 overexpression have a growth advantage under long-term treatment with relapse-permissive doses of vincristine. All cells were treated for 12 days while in co-culture with OP9 stromal support. Cell proliferation, measured by an assay for ATP levels, is expressed as a percentage of the PBS control at each time point. **(A–C)** US7, LAX57, and LAX56 cells as indicated and treated with 0.75 nM, 2.5 nM, or 5 nM vincristine. **(A)** Mean ± SEM of *n* = 8 replicates per time point per sample combined from two independent experiments. **(B, C)** Mean ± SEM of *n* = 4 replicates per time point per sample. Two-way ANOVA, adjusted *p*-values, Šidák’s multiple comparison test. *p < 0.05, ***p < 0.001, ****p < 0.0001.

*In vivo*, increased ST6Gal1 expression stimulated growth of BCP-ALL cells compared to cells with lower expression levels. We then examined if this could be recapitulated in a two-dimensional tissue culture model. This system makes use of co-culture with mitotically inactivated OP9 stromal cells to support growth and viability of the leukemia cells. However, under steady-state conditions, proliferation of US7-ST6Gal1 OE and EV cells was comparable (**Supplementary Figure 3A**). We also treated the cells with vincristine. As shown in **Figure 5A**, when treated with a suboptimal [non-lethal] dose of vincristine, after prolonged exposure to the drug, US7 cells with increased expression of ST6Gal1 maintained higher viable cell numbers compared to the control. Since US7 cells were from a patient at diagnosis, we also tested a second diagnosis BCP-ALL, LAX57, as well as a relapse sample, LAX56. Increased expression of ST6Gal1 in LAX57 and in LAX56 (**Supplementary Figure 1A**) also promoted resistance to vincristine, although in LAX57, the difference with control cells was less than that in LAX56 (**Figures 5B, C**).

### BCP-ALL Cells With Knockdown of ST6Gal1 Expression Also Are More Vincristine Resistant

**Figure 6:**
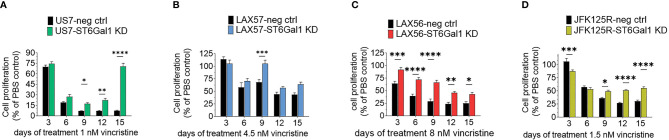
Knockdown of ST6GAL1 expression correlates with increased resistance to vincristine. Paired sets of control BCP-ALLs and cells with ST6Gal1 knockdown were plated on mitotically inactivated OP9 cells and treated for 15 days with the indicated concentrations of vincristine. **(A, D)** Values mean ±SEM of *n* = 3–4 replicates per time point per sample. **(B, C)** Four to six replicates per time point per sample. Two-way ANOVA, Šidák’s multiple comparison test, adjusted *p*-values, *p < 0.05, **p < 0.01; ***p < 0.001, ****p < 0.0001.

We also reduced ST6Gal1 expression in US7 and LAX57 as diagnosis samples, and in LAX57 and JFK125R as relapses (**Supplementary Table 1**) using Cas9/CRISPR gene editing. FACS using SNA lectin was used as readout and selection method for tracking ablation of *ST6GAL1* gene function through loss of α2,6 sialylation (**Supplementary Figure 1B**). Western blotting also confirmed substantial reduction in ST6Gal1 protein levels (**Supplementary Figure 1C**). Because sialylation of the lysosomal/cell surface protein Lamp1/CD109a was reported to stimulate lysosomal exocytosis (33), we also specifically investigated the degree of α2,6 sialylation of Lamp1 in the *ST6GAL*1 knockdown cells using a SNA affinity column. As shown in **Supplementary Figure 1D**, whereas Lamp1 protein isolated from wild-type cells bound to the SNA affinity column, knockdown of ST6Gal1 largely eliminated the ability of Lamp1 to be retained on the column. Thus, reduction of ST6Gal1 activity was clearly achieved in these BCP-ALLs. Steady-state growth of these cells in the absence of drug treatment was not consistently affected (**Supplementary Figure 4**). We also tested the four different BCP-ALLs with reduced ST6Gal1 levels in a long-term co-culture with OP9 cells for sensitivity to vincristine. As shown in **Figure 6**, cells expressing lower levels of ST6Gal1 were, to a varying degree, more tolerant to vincristine treatment than the matched original wild-type cells. We conclude that in *in vitro* co-culture, neither ST6Gal1 overexpression nor knockdown consistently affects steady-state proliferation of these BCP-ALL cells but changes in ST6Gal1 expression levels do reduce the ability of the cells to respond to the stress of vincristine drug treatment.

### Effect of Stromal Galectin-1 on BCP-ALL Cells With ST6Gal1 Overexpression

**Figure 7:**
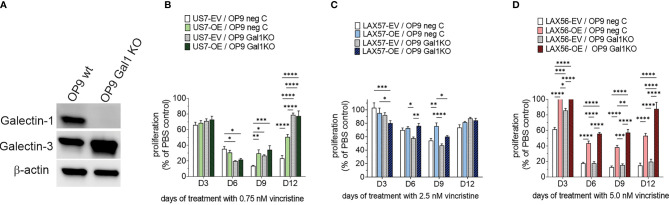
Effect of co-culture of BCP-ALL cells on OP9 stromal cells lacking Galectin-1. **(A)** Western blot documenting loss of Galectin-1 in selected OP9 *Galectin-1* ko clone. **(B–D)** US7, LAX57, or LAX56 cells as indicated grown on control OP9 (OP9 neg C) or OP9 *Galectin-1* knockout (Gal1 KO) cells. US7, LAX57, and LAX56 were treated with 0.75, 2.5, and 5 nM vincristine. Values, mean ±SEM of *n* = 4 replicates per time point per sample. Two-way ANOVA, Tukey’s multiple comparison test, adjusted *p*-values. *p < 0.05, **p < 0.01; ***p < 0.001, ****p < 0.0001.

A relatively well-described consequence of the sialylation of glycoproteins on the cell surface is to allow or inhibit the binding of lectins, a type of protein that specifically recognizes and binds to carbohydrates. Galectin-1 is such a lectin and it is inhibited in its binding to client glycoproteins by their α2-6 N-linked sialylation (34). Glycan–Galectin interactions are known to regulate B-cell function (35) and Galectin-1 plays a role in immune modulation as well as in cancer (36–38). Our previous studies had shown that inhibition of Galectin-1 using a drug, PTX008, sensitizes BCP-ALL cells to chemotherapy (39). These BCP-ALL cells endogenously produce Galectin-1 to a varying degree (39), but stromal cells can also be a source of extracellular Galectin-1 (40). Therefore, we knocked Galectin-1 out in the OP9 stromal cells used for co-culture, *via* Cas9/CRISPR (**Figure 7A**), and tested the effect on BCP-ALL cell growth and resistance to vincristine treatment in co-culture with the knockout cells. We found that wild-type and Galectin-1 knockout OP9 cells supported wild-type and ST6Gal1 OE US7 cells equally well under normal growth conditions (**Supplementary Figure 3B**), excluding a major role for stromal Galectin-1 interactions with cell surface glycoproteins that are sialylated by ST6Gal1 during normal growth. After 12 days of vincristine chemotherapy, proliferation of BCP-ALL cells with original levels of ST6Gal1 expression (EV samples **Figures 7B–D**) plated on OP9 Galectin-1 knockout stroma was comparable (LAX57 and LAX56) or enhanced (US7) (**Figures 7B, C**, compare white bars) with respect to the same cell types grown on wild-type OP9 cells. On day 12, US7 ST6Gal1 OE and LAX56 ST6Gal1 OE cultures grown on OP9 Galectin-1 knockout cells (**Figures 7B, D**) also had higher cell counts. Based on literature data, increased glycoprotein sialylation by ST6Gal1 should reduce Galectin-1 binding. Based on our PTX008 inhibitor studies (39), reduced Galectin-1 binding in turn should chemo-sensitize the BCP-ALL cells. Instead, OP9 Galectin-1 knockout cells protected BCP-ALL cells as well as, or better than, WT cells (**Figures 7B–D**). Thus, stromal-produced Galectin-1 binding to α2,6 N-glycoproteins on BCP-ALL cells is not mechanistically linked to the enhanced resistance of ST6Gal1 OE cells to vincristine stress.

### Increased Expression of ST6Gal1 Associates With Relatively Large Transcriptome Changes

**Figure 8:**
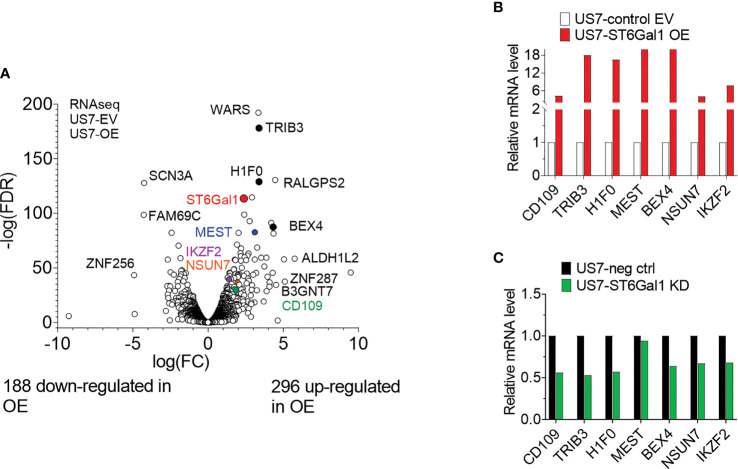
Transcriptome of US7 cells with different levels of ST6Gal1 expression. **(A)** Volcano plot summarizing 484 differentially expressed genes (>2-fold; *p* > 0.05, rpkm cutoff = 1) in BCP-ALL cells with increased ST6Gal1 levels, with approximately 60% of protein-encoding expressed genes showing up-regulation. *n* = 3 biological replicates per RNA sample. **(B)** Real-time RT-PCR on selected genes with increased expression in the US7 ST6Gal1 OE cells. Values for the control EV samples were set as 1 and results are expressed as fold change. Note the discontinuity of the *Y*-axis. **(C)** Comparison of expression of selected genes in matched US7 control and US7 ST6Gal1 knockdown cells using real-time RT/PCR.

In other types of cancer cells, ST6Gal1 expression was reported to regulate transcription [e.g., (41)]. We therefore also compared the transcriptomes of US7 ST6Gal1 OE and EV control cells. As expected, *ST6GAL1* RNA was significantly increased in the US7 ST6Gal1 OE cells (**Figure 8A** and **Supplementary Table 2**). In addition, we found differential expression of approximately 5% of all the protein-encoding genes that are expressed in these cells (**Supplementary Table 3**). Schultz et al. (42) previously reported that increased ST6Gal1 expression correlates with increased expression of the stem cell transcription factor Sox9 in colon and pancreatic cancer cell lines, conferring a stem-cell-like phenotype. However, in the BCP-ALL cells studied here, the gene expression data did not point to induction of a more stem-cell-like or primitive phenotype with increased *ST6GAL1* expression. Instead, Ingenuity Pathway Analysis of the US7 OE/EV RNA-seq data indicated “increased neoplasia” of the US7 ST6GAL1 OE cells compared to cells with baseline levels of *ST6GAL1* (**Supplementary Table 2**). We therefore compared differential gene expression in ST6Gal1 overexpressing US7 cells with that of a matched set of 10 diagnosis/relapsed BCP-ALL samples (43). Interestingly, there were 29 genes with common differential expression, of which 19 were regulated in the same direction in US7 ST6Gal1 OE cells and relapses, including VEGFA and TGFβ2 (**Supplementary Table 2**).

In terms of drug resistance in *in vitro* co-culture, we compared our data to ICN13 BCP-ALL cells that had been treated with relapse-permissive doses of vincristine while in co-culture with OP9 cells (Oliveira et al., in preparation). In that study, on d30 of drug treatment, 948 genes were differentially expressed compared to PBS-treated controls cultured for the same period of time. A comparison of the transcriptome of ST6Gal1 overexpressing US7 cells with vincristine-resistant ICN13 cells showed overlap of 78 genes with differential expression (**Supplementary Table 3**, **Supplementary Figure 5A**). However, 69 of these showed an increase in one condition (ICN13 × vincristine) and decrease in the other (ST6Gal1 overexpression), ruling out a straightforward positive correlative effect for specific genes that would account for increased *in vitro* vincristine resistance in US7 cells with increased ST6Gal1 expression.

Real-time RT/PCR was used to further validate increased mRNA levels of six selected genes in US7 ST6Gal1 OE cells. These included CD109 and BEX4, two genes that had high expression in MLL-r samples compared to normal pre-B controls (25). CD109 was of interest because increased expression correlates with worse outcome in AML and diffuse large B-cell lymphoma (44, 45). The stress pseudo-kinase TRIB3 is also implicated in acute leukemias (46, 47), and IZKF2 is a well-known transcription factor in normal and malignant hematopoietic cells (48, 49). As shown in **Figure 8B**, the analysis validated higher expression of these genes in US7 ST6Gal1 overexpressing cells. Conversely, expression of the genes was somewhat lower in ST6Gal1 knockdown US7 cells (**Figure 8C**). However, real-time RT/PCR analysis for expression of the same genes in LAX56 and LAX57 with ST6Gal1 overexpression did not yield a similar outcome (**Supplementary Figure 6A**). In addition, ST6Gal1 knockdown in two additional BCP-ALLs, ICN13 and BM41 (**Supplementary Table 1**, **Supplementary Figure 6B**) did not provide results consistent with those in US7 cells, ruling out a universal regulation of these genes by ST6Gal1 expression in BCP-ALL. Therefore, we did not find consistent changes in different BCP-ALLs in the expression of protein-encoding genes that could correlate with levels of ST6Gal1 and would explain the increased ability of ST6Gal1 overexpressing cells to proliferate in mice, and their increased resilience against vincristine stress *in vitro* co-culture.

## Discussion

### ST6Gal1, a Non-Essential Protein With Unique Enzymatic Activity, as an Attractive Target for Treatment of Leukemias?

Although ST6Gal1 is thought to be the main enzyme responsible for the bulk of N-glycoprotein-linked α2-6 sialylation, mice with total *St6gal1* knockout are viable, with a surprisingly mild phenotype mainly manifest in immune cell function: increased inflammation, defects in dendritic cell, and myelopoiesis, as well as mature B-cell development (50–52). Also, the phenotype of mice with specific knockout of S*t6gal1* in the liver, an organ with particularly high ST6Gal1 expression, is mild (32). Thus, as a possible therapeutic target, ST6Gal1 would be attractive if increased expression is causally related to features associated with a more malignant phenotype. Indeed, as reviewed (53), numerous studies correlate ST6Gal1 overexpression with some aspects of increased malignancy in other cancers [also (41)].

A possible contribution of ST6Gal1 to hematological malignancies has been much less well-studied. The exception is multiple myeloma, in which ST6Gal1 secreted by more mature B cells in the bone marrow suppressed myeloid development (54). It is important to note in this context that normal hematopoietic progenitor stages express different levels of *ST6GAL1* mRNA, with low expression in hematopoietic stem cells, which progressively increases during maturation along the B-lineage (**Figure 2**). Thus, the varying expression levels of *ST6GAL1* in different BCP-ALL subtypes noted here may also, in part, be normal for the stage at which the cells have become arrested in their maturation.

However, we noted that *ST6GAL1* expression in the more than 20 subtypes of leukemic B cell precursors that have currently been distinguished (1) varied widely even within a specific subgroup. Accordingly, although in pediatric ALL, high *ST6GAL1* expression correlated with better relapse-free survival and relapsed samples had lower expression (**Supplementary Figures 7A, B**), in adult ALL, the overall survival probability was in fact similar [at *p* = 0.37, ns] for patients with high *ST6GAL1* (**Supplementary Figure 7D**). In addition, adult patients who achieved a complete remission had lower *ST6GAL1* mRNA than those who did not (**Supplementary Figure 7E**). Thus, in hematopoietic malignancies, there is no clear-cut correlation between *ST6GAL1* expression and clinical outcome.

### Non-Concordant Phenotype of ST6Gal1 Overexpression and Knockdown in BCP-ALL Cells *In Vitro* and *In Vivo*

Frequently, the importance of a gene for a biological process is evaluated by loss-of-function and/or gain-of-function experiments; typically, this entails knockout/knockdown and overexpression. We used overexpression to investigate if increased ST6Gal1 levels in BCP-ALL contribute to a more malignant phenotype in mice. In this system, overexpression in US7 cells clearly promoted increased malignancy, in the sense that the overexpressing cells proliferated more rapidly than the cells with baseline expression, which was also seen after cessation of vincristine treatment. However, in tissue culture, there was no consistent effect of ST6Gal1 expression levels on proliferation rate. This suggests somewhat unsurprisingly that, *in vivo*, some interactions of the BCP-ALL cells with the microenvironment are not recapitulated in the tissue culture model. For example, based on the reported suppression of myeloid development by ST6Gal1 in multiple myeloma (54), it is possible that ST6Gal1 overexpressing BCP-ALL cells suppressed myeloid development in the bone marrow, which, in turn, could promote leukemia proliferation.

In contrast, in tissue culture, high ST6Gal1 contributed statistically significantly to increased drug insensitivity in three different BCP-ALLs. However, unexpectedly, in all three BCP-ALLs, ST6Gal1 knockdown also decreased responsiveness to chemotherapy, suggesting a complex contribution of ST6Gal1 to this process. Based on these findings, we posit that effects of different *ST6GAL1* expression levels in BCP-ALL are unlikely to be captured in a simple gain-of-function/loss-of-function dichotomy. We hypothesize that this could be explained by the inherent nature of the enzymatic activity of this protein, as detailed below.

### Expression Levels of *ST6GAL1* mRNA May Not Correspond to Levels of N-Linked α2,6 Sialylation

There is no linear correlation between the expression of the *ST6GAL1* mRNA, and the generation of specific sialylation on glycoproteins: as with many other glycosyltransferases, ST6Gal1 does not function in 1:1 stoichiometry with client proteins since it can attach one or multiple sialic acids to a single glycoprotein. Indeed, Oswald et al. (32), who studied mice with liver-specific *St6gal1* knockout, remarked “our findings demonstrate that transcriptional changes, or lack thereof, cannot be reliably used as a surrogate for regulated changes in protein glycosylation within a cell”. In addition, the sialylation of glycan structures is determined not only by ST6Gal1 protein levels but also by hypoxia (55), interactions of ST6Gal1 with the glycosyltransferase B4Galt1 (56), and metabolic flux (57, 58), which can regulate the availability of the donor sialic acid. The existence of inherent variability in sialylation is supported by other studies in which we analyzed the glycome of US7, LAX56 and LAX57 EV, and ST6Gal1 OE cells (Oliveira et al., in preparation). In a different study, we analyzed the glycome of drug-resistant ICN13 BCP-ALL cells and found that these cells exhibit reduced overall sialylation, with a shift from α2-6- to α2-3-linked Sia without significant changes in expression of *ST6GAL1* (Oliveira et al., in preparation). These results may partly explain the inconsistent phenotypes found here associated with different ST6Gal1 expression levels.

### Expression of Specific Glycoprotein Clients of ST6Gal1 N-linked α2,6 Sialylation, and the Impact of Each of These Clients on BCP-ALL Proliferation and Vincristine Resistance May Vary in Different BCP-ALL Samples

In some carcinomas, glycoproteins such as the EGFR and ErbB2 function as critical oncogenes that consistently drive the tumor phenotype. Interestingly, ST6Gal1 sialylation of these receptors was linked to sensitivity to cetuximab and trastuzumab therapeutic monoclonal antibodies (59, 60). Unfortunately, whether BCP-ALL cells of all subtypes and at different stages of treatment (diagnosis and relapse) consistently express one or more of such glycoproteins, of which the α2,6 N-linked sialylation would be critical for cell growth or drug resistance, remains unknown. Seeing that more than 350 ST6Gal1 client glycoproteins have been identified in different cell types (17–19), identification of such a putative critical glycoprotein, if there is one, is complicated. Moreover, B-lineage leukemias represent a continuum of differentiation stages and not all glycoproteins are expressed at every stage. CD75 is an example of an epitope generated by ST6Gal1 (61) which apparently is not expressed on BCP-ALL cells but is present on normal peripheral blood CD19+ B-cells (**Supplementary Figure 8**). A recent report documenting the existence of N-linked sialylated RNAs further adds to the potential complexity of ST6Gal1 involvement (62).

### A Relatively Large Effect of ST6Gal1 Overexpression on Transcriptome Is Consistent With a General, Broad Effect of N-Linked α2,6 Sialylation on BCP-ALL Physiology

Apart from increased malignancy, pathway analysis of our RNA-seq data showed a correlation between increased *ST6GAL1* expression and a reduced migration and adhesion profile of the cells (**Supplementary Table 2**). We note that this correlation was unexpected in view of the lack of difference between US7 OE and EV cells in the *in vivo* bone marrow homing assay (**Figure 3A**). Moreover, the more drug-resistant phenotype of the ST6Gal1 OE cells suggests that they should have superior migration and adhesion to protective stromal cells (63, 64). However, it is consistent with the functional assay by Woodard-Grice et al. who overexpressed ST6Gal1 in acute myelogenous leukemia cell lines and found decreased α4β1-mediated VCAM1 binding (65). Thus, it is possible, based on changes in RNA expression, that other glycoproteins that can be sialylated by ST6Gal1 such as VEGFA (66) contribute to this complex phenotype.

Overall, our gene expression analysis in which we found differential expression of 484 genes in fact supports a broad effect of ST6Gal1 overexpression on the transcriptome, consistent with involvement of multiple glycoproteins and multiple downstream effects. The finding that increased ST6Gal1 expression also changes levels of mRNAs encoding its own substrate proteins adds further complexity (**Supplementary Figure 5B**). Interestingly, variability was also reported by Venturi et al. (16) who found that increased ST6Gal1 levels caused very large transcriptome changes in one but not in a different colon cancer cell line. Surprisingly, in view of the very different cell types, we found that US7 cells with ST6Gal1 overexpression had 19 genes in common (18 increased and one decreased) with the SW948 ST6Gal1 overexpressing colon cancer cells, including, among others, *ST6GAL1*, *TGFβ2*, and *CTF1*.

### Conclusion

Venturi et al. (16) who investigated colon cancer cell lines stated that “changes induced by ST6Gal1 expression … are strongly cell-type specific, ruling out that the association of ST6Gal1 and malignancy is a general paradigm”. Our studies support this concept, and furthermore indicate that ST6Gal1 in BCP-ALL is neither an oncogene nor a tumor suppressor. This does not exclude an important contribution of ST6Gal1 to the outcome of specific therapies such as those making use of monoclonal antibodies, as described for the EGFR and ErbB2 (59, 60). However, detailed analytical glycan studies of sialylation on CD19, CD22, or CD20 glycoproteins before and after treatment with antibodies or CAR T-cells would be needed to determine if ST6Gal1 N-linked α2,6 sialylation is a contributing factor to resistance in B-cell malignancies treated with such immune therapies.

## Materials and Methods

### Cell Culture and Drug Treatment

GSE102301 describes that US7 [LAX7] and US7R [LAX7R] were obtained from a patient at diagnosis and after relapse following a standard 3-week chemotherapy regimen (vincristine, dexamethasone, L-asparaginase, and doxorubicin). JFK125/JFK125R, SF06/SF06R, and US7/US7R PDX patient-derived pre-B ALL samples have been previously described (67, 68). LAX56 and LAX57 grew directly on OP9 cells and have also been previously described (69). These BCP-ALLs are all largely stromal-dependent and were grown in co-culture with mitotically inactivated OP9 bone marrow stromal cells (ATCC CRL-2749). They were STR genotyped to confirm their identity. OP9 cells allowed to adhere overnight were treated with 10 µg/ml mitomycin C (Sigma, Cat#M4287) for 3 h in complete medium, washed, and used for co-culture with human ALL cells. Cells were co-cultured in α-MEM media supplemented with 20% FBS, 1% L-glutamine, and 100 μg/ml penicillin/streptomycin (Life Technologies, Grand Island, NY). All cell lines used are listed in **Supplementary Table 1**. RS4;11 was obtained from the ATCC. Glycan analysis was performed as described previously (25).

For *in vitro* drug treatment, cells were plated at 0.5 × 10^6^ cells/well in a 24-well plate with an OP9 feeder layer. Vincristine sulfate (Sigma, Cat#V8388) diluted in PBS at different concentrations was added freshly every 3 days. Each different BCP-ALL was titrated with different concentrations of vincristine to identify concentrations that would significantly inhibit proliferation but not eradicate all leukemia cells. Vincristine stocks were stored in small aliquots at −80°C. Diluted samples stored at 4°C were used within 14 days. Cell viability was determined on cells migrated into the tissue culture medium using a CellTiterGlo viability assay (Promega, Cat#G7570) according to the manufacturer’s instructions.

### Lentiviral Constructs and Transduction

The empty pLV411G vector was obtained from Simon Barry (70). pLV411G-ST6Gal1 was obtained from Dukka Škalamera (71). Inserts were introduced into the pLV411G vector by GateWay cloning (Invitrogen). The insert encodes the human ST6Gal1 406 amino acid isoform A, which we verified by DNA sequencing, in addition to a small C-terminal extension due to the cloning procedure. 293FT cells were cultured in high-glucose Dulbecco’s modified Eagle’s medium (DMEM, Gibco, Cat# 11995073) with 10% fetal bovine serum (FBS, Atlanta Biologicals, Cat# S11150H, Lot# K18135), 100 IU/ml penicillin and 100 μg/ml streptomycin (Gibco, Cat# 15070063). Lentiviral supernatant was produced by co-transfecting HEK 293FT cells with the plasmids pCD/NL-BH*DDD, pCMV-VSV-G (from AddGene), and pLV411G (with or without human *ST6GAL1*) using Lipofectamine 2000 (Invitrogen, Cat# 11-668-019) in Opti-MEM (Invitrogen) medium. The culture medium with the DNA/lipofectamine mixture was replaced after 3–4 h by DMEM medium with 10% FBS. After incubation overnight, the medium was replaced with DMEM medium containing 10% FBS and 10 mM sodium butyrate. After incubation for 6–8 h, the medium was replaced with regular growth medium. Twenty-four hours later, lentiviral supernatant was collected, filtered through a 0.45-μm filter, and loaded by centrifugation (600*g*, 30 min at 32°C) onto non-tissue culture six-well plates coated with 50 μg/ml RetroNectin (Takara). The LV backbone also encodes green fluorescent protein (GFP), which was used for flow-sorting of transductants on a BD Aria Fusion flow cytometer. LAX56 and LAX57 were transduced with the same LV vector for ST6Gal1 overexpression, but with a different empty vector control—pCL6IEGWO-GPF. All transductants were purified using flow sorting. US7 cells were also transduced with pCL6IEGWO-blasto-luc, a luciferase LV vector and selected with 8 μg/ml blasticidin, after a pilot of 4–20 μg/ml in a 6-day assay to determine a suitable selection concentration.

### Cas9/CRISPR Knockout Conditions for *ST6GAL1*

For gene deletion in BCP-ALL cells, predesigned crRNAs, non-targeting control guide RNAs, trRNAs, and Cas9 protein were purchased from Integrated DNA Technologies (IDT, Coralville, Iowa). The same guide RNA against human *ST6GAL1* (IDT Hs.Cas9.ST6GAL1.1.AC; position 187072904 with the sequence CAGATGGGTCCCATACAATT AGG) was used for the different pre-B ALLs. Alt-R^®^ CRISPR-Cas9 guide RNA for human *ST6GAL1* (crRNAs, 100 μM) and Alt-R^®^ CRISPR-Cas9 tracrRNAs (trRNAs, 100 μM) were annealed by incubation at 95°C for 5 min. After cooling to room temperature, Alt-R^®^ S.p. HiFi Cas9 Nuclease 3NLS (recombinant Cas9 protein, 1 μg/μl) was then added to the RNA mixture and RNA ribonucleoprotein complexes were allowed to form for 20 min. Electroporation of approximately 5 × 10^6^ cells in Neon buffer T was performed using 3 pulses at 1, 600 V for 10 ms each on a Neon transfection system (Thermo Fisher) with the addition of 10 nmol Alt-R^®^ Cas9 Electroporation Enhancer. Twenty-four hours after electroporation, fresh culture medium was added.

### OP9 Galectin-1 Knockout

We combined two guide RNAs against mouse Galectin-1 (IDT Mm.Cas9.LGALS1.1.AA; position 78929743 with the sequence GACCTGGGGAACCGAACACC GGG and IDT Mm.Cas9.LGALS1.1AB position 78928002 with the sequence CGAACTTTGAGACATTCCCC AGG) to target Galectin-1 in OP9 cells. A total of 2 × 10^6^ cells in Neon buffer T were electroporated using one pulse at 1,350 V for 30 ms as described above. Galectin-1 knockdown was confirmed by Western blot 72 h after electroporation. To isolate Galectin-1 knockout cells, single cells were sorted on a BD Aria Fusion around day 14 after electroporation. Single clones in 96-well plates were continuously expanded for 4 weeks with medium change weekly after the first 2 weeks of culture. Thereafter, growing clones were transferred to 24-well plates and then to 6-well plates. Galectin-1 knockout clones were verified by Western blotting and viably stored in LN_2_.

### Monitoring of *ST6GAL1* Gene Disruption by FACS Using SNA

Knockdown of ST6Gal1 was monitored using FACS for *Sambucus nigra* (SNA) cell surface reactivity on live cells. Careful titration of the amount of SNA lectin used for sorting was needed because exposure of the cells to high concentrations of SNA resulted in cell death. This is due to the fact that SNA I, which was obtained from Vector labs (Cat #B-1305), is a chimeric lectin composed of an A-chain with enzymatic activity and a B-chain with carbohydrate-binding activity. The A-chain encodes a ribosome-inactivating protein (72). BCP-ALL cells were blocked with human FCR blocking reagent diluted 1:100 (MACS Miltenyi Biotec, Cat#130-059-901) for 15 min at 4 °C. Cells were then incubated for 15 min at 4 °C with biotinylated SNA lectin diluted 1:100 followed by 15 min at 4 °C with streptavidin-APC diluted 1:200 (eBioscience, Cat# 17-4317-82). DAPI was added at a 1:100 dilution to distinguish dead and live cells. To enrich for *ST6GAL1* knockdown cells, we flow sorted cells on a BD Aria Fusion X20 around day 10 after electroporation. For some ALLs, electroporation with sgRNA was done twice. Using these procedures for example on day 5 after a single electroporation, there were 95.5% SNA^med^ and 0.12% SNA^neg^ cells in the LAX56 population, whereas for LAX57, this was 82.5% SNA^med^ and 1.3% SNA^neg^ cells. Repeat of the electroporation and flow sorting of the SNA^med/neg^ cells failed to further yield pure SNA^neg^ populations for any of the BCP-ALLs (**Supplementary Figure 1B**).

### Western Blotting

For Western blots for ST6Gal1 protein, cells were lysed in RIPA buffer with added protease and phosphatase inhibitors. We used R&D Systems human ST6Gal1 antibody diluted 1:500 (Cat#AF5924) and β-actin as loading control (Santa Cruz, 1:500, Cat#sc-47778 HRP). We also assessed the effect of ST6Gal1 ablation on Lamp1 α2,6 sialylation. BCP-ALL cells were lysed in Triton T-100 lysis buffer with glycerol at pH 7.4 (Alfa Aesar, Cat#J63866AK) and glycoproteins were captured with SNA-biotin (Vector labs Cat #B-1305). Dynabeads Streptavidin magnetic beads (Invitrogen, Cat#65801D) were used to isolate the SNA-bound glycoproteins. Proteins were separated on 4%–20% SDS-PAA gradient gels (Mini-PROTEAN^®^ TGX Stain-Free™ Protein Gels, Bio-Rad, Cat#4568094). Lamp1 (CD109a) antibodies used at 1:1,000 dilution were from BioLegend (Cat#328602). The WB for OP9 cells used anti-Galectin-3 (BioLegend, 1:1,000, Cat#125402) or Galectin-1 (R&D Systems, 1:1,000, Cat#AF1152) antibodies. Western blotting for α2,6-sialylated proteins made use of biotinylated SNA from Vector Laboratories.

### Mouse Experiments

For bone marrow homing experiments, 10^7^ cells were injected *via* the tail vein into NSG mice (*n* = 3–4 per group). Sixteen hours later, bone marrows were analyzed by FACS for CD19, CD10, and eGFP-positive cells. Results are expressed as cell percentage in the live cell lymphocyte gate. To measure survival, non-irradiated NSG mice 8–10 weeks of age were used in all experiments. Female [*n* = 5 for US7/EV and *n* = 7 US7/OE] or male mice [*n* = 5 per group] were injected with 2 × 10^6^ leukemia cells on d0. Imaging for leukemia signal was performed once per week by i.p. injection of 2.5 mg of D-luciferin in 200 μl of PBS. End points included loss of >20% initial body weight. For vincristine treatment, we used *n* = 5 female mice per group. Mice received six weekly vincristine treatments [0.5 mg/kg; i.p.] starting on d14. Bioluminescence signals were quantified using Aura imaging software (Spectral Instruments Imaging, LLC Tucson, AZ).

All animal experiments were conducted under an IACUC-approved institutional protocol. Methods of euthanasia were consistent with the guidelines of the American Veterinary Medical Association.

### RNA Expression Analysis

RNAs were isolated from the cells by Trizol extraction. RNA-seq was performed by Novogene using an unstranded high-throughput TruSeq stranded mRNA prep kit. Analysis of the RNA-seq data was performed as previously described (25). The genome build used for analysis was hg38 and 19,862 protein-encoding genes were included in the analysis. Significantly regulated genes were defined as fold change ≥2, *p* < 0.05, and low expression filter set at rpkm <1.0. Graphs showing normalized RNA counts were generated using GraphPad Prism (v8.4.3). QIAGEN Ingenuity Pathway Analysis (IPA) version 62089861 was used to analyze results of RNA-seq for pathways with differential regulation using rpkm>1, *p*-values and FDR at <0.05 and logFc at −1.0 to 1.0. RNA-seq data were deposited in GEO under accession number GSE185611. Accession to all data is listed in **Supplementary Table 4**.

For real-time RT/PCR, RNA was extracted using an RNeasy Plus Mini Kit (Cat# 74134, QIAGEN). A high-capacity cDNA reverse transcription kit was from ABI (Cat# 4368814). cDNA concentrations were determined by Nanodrop. The Power SYBR™ Green PCR Master Mix was purchased from Life Technologies (Cat# 4367659). PCR was on an ABI QuantStudio 7 Flex System with 40 cycles and anneal/extend temperature set at 60°C.

Primers obtained from IDT (Integrated DNA Technologies) included the following:

**Table:** 

Gene	Forward Primer	Reverse Primer
hCD109	AAGCCAGTGAAAGGAGACGTA	CCAGGGGAAGATAGATCCAGG
hTRIB3	AAGCGGTTGGAGTTGGATGAC	CACGATCTGGAGCAGTAGGTG
hH1F0	ACTCGCAGATCAAGTTGTCCA	GGTTCGTCGCTCTTGGCTA
hMEST	ATCGGGTGATTGCCCTTGATT	GAAAGAAGGTTGATCCTGCGG
hBEX4	AAAGAGGAACTAGCGGCAAAC	CCAAATGGCGGGATTCTTCTTC
hNSUN7	GGACTCCGTTTATGTCATGGC	CTCAGACTCGGACAAGGACC
hIKZF2	AACTACTGTGGACGAAGCTACA	CGTTTTCCCATATTCCCCGTG
hActin	CATGTACGTTGCTATCCAGGC	CTCCTTAATGTCACGCACGAT

### Data Availability and Statistical Analysis

The origin and availability of the data analyzed here are summarized in **Supplementary Table 4**. Results were analyzed statistically using GraphPad Prism 8.3.1 and Excel software. The value of *p* < 0.05 was considered statistically significant. Details of biological replicate numbers and statistical tests used to analyze significance are indicated in each figure legend.

## Data Availability Statement

The datasets presented in this study can be found in online repositories. The names of the repository/repositories and accession number(s) can be found in the article/**Supplementary Material**.

## Ethics Statement

The animal study was reviewed and approved by the City of Hope (COH) Institutional Animal Care and Use Committee (IACUC).

## Author Contributions

MZ, TQ, DK, and NH: conceptualization. LY and NH: data curation. MZ, TQ, and LY: formal analysis. NH and DK: funding acquisition. MZ and TQ: investigation. NH: project administration. NH and DK: supervision. NH: writing. All authors contributed to the article and approved the submitted version.

## Funding

This study was partly supported in 2016/2017 by a New Idea Award from the Leukemia Lymphoma Society and NIH R01 CA172040 and CA090321 to NH. DK was supported in part by Civic Solutions Inc and is the recipient of an Australian Research Council Future Fellowship (project number FT160100344) funded by the Australian Government. Research reported in this publication included work performed in the City of Hope Small Animal Studies Core supported by the National Cancer Institute of the National Institutes of Health under award number P30CA033572. The content is solely the responsibility of the authors and does not necessarily represent the official views of the National Institutes of Health. The funders were not involved in the study design, collection, analysis, interpretation of data, the writing of this article or the decision to submit it for publication.

## Conflict of Interest

The authors declare that the research was conducted in the absence of any commercial or financial relationships that could be construed as a potential conflict of interest.

## Publisher’s Note

All claims expressed in this article are solely those of the authors and do not necessarily represent those of their affiliated organizations, or those of the publisher, the editors and the reviewers. Any product that may be evaluated in this article, or claim that may be made by its manufacturer, is not guaranteed or endorsed by the publisher.

## Supplementary Material

Supplementary Table 2 US7 OE EV ST6Gal1 RNA-seq data.

## Abbreviations

BCP-ALL, B-cell precursor acute lymphoblastic leukemia; GEP, gene expression profiling; rpkm, reads per kilobase million.
