# Supplementary material for: Multi-Faceted Effects of ST6Gal1 Expression on Precursor B-Lineage Acute Lymphoblastic Leukemia
Source: Front Oncol. 2022 Mar 16;12:828041. doi: 10.3389/fonc.2022.828041 (PMC8967368; doi:10.3389/fonc.2022.828041)
Supplement: Supplementary file 1 [file DataSheet_1.pdf]

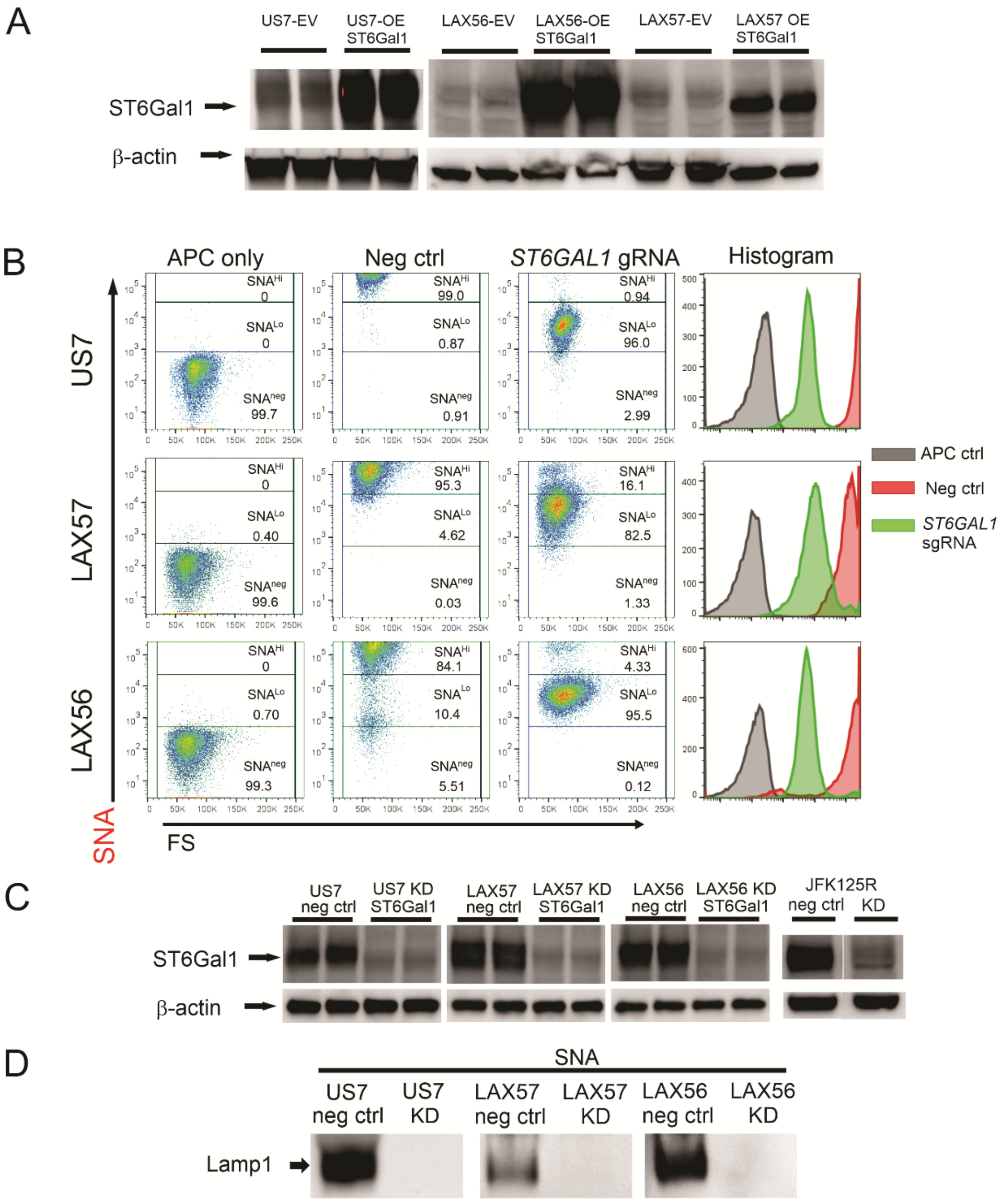

Supplementary Figure 1. Modulating ST6Gal1 levels by lentiviral transduction and Cas9/CRISPR gene editing. (A) Western blot analyses for overexpression of ST6Gal1. β-actin, loading control. EV, empty vector-transduced cells; OE, overexpressing cells transduced with a lentiviral vector encoding human ST6Gal1. Samples were loaded in duplicate wells. **(B)** Monitoring of *ST6GAL1* knockdown using *Sambucus nigra (*SNA) lectin and FACS on the indicated BCP-ALLs. APC only: streptavidin-APC, no biotinylated SNA added. Neg control, input BCP-ALL cells without knock down. Input cell populations: mostly SNA-positive with high signal. SNA recognizes Sia attached to terminal Gal residues in an α2-6 linkage. Percentages indicate cells falling into different categories of SNA reactivity. (**C**) Western blot analyses of ST6Gal1 expression. RIPA buffer cell lysates with loading in duplicate wells. β-Actin, loading control. (**D**) Lamp1 α2,6 sialylation in *ST6GAL1* knockdown BCP-ALL cells. SNA-biotin binding glycoproteins were purified using streptavidin-conjugated magnetic beads from the indicated cells followed by Western blotting for Lamp1.

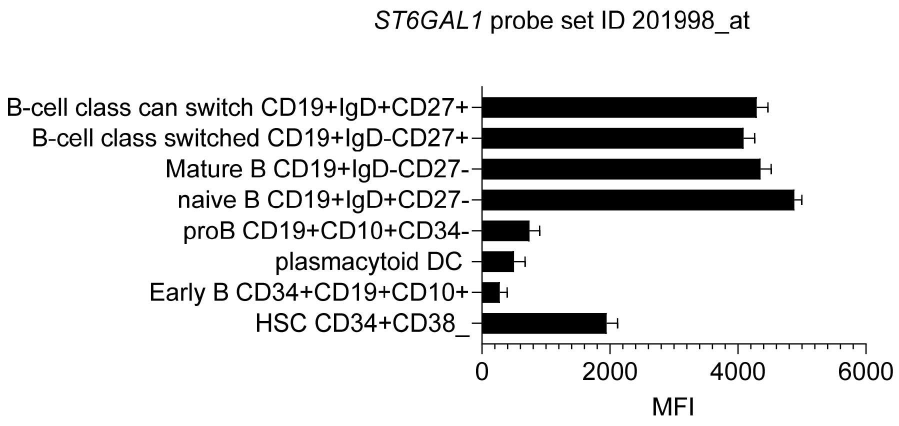

Supplementary Figure 2. Expression of *ST6GAL1* mRNA increases during human B-cell development. Data downloaded from BloodSpot (ku.dk) . Purified human hematopoietic cells identified by the indicated cell surface markers. n=3-5 samples per cell type. Log-transformed GEP intensity values, Affymetrix genome arrays. Original data GSE24759 (PMID 21241896).

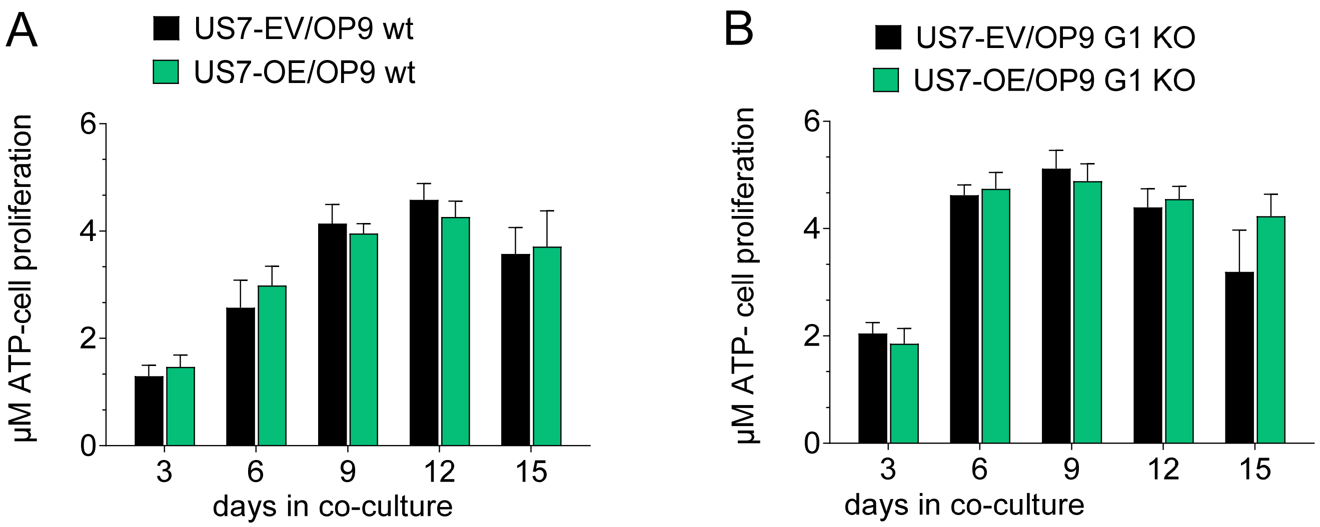

Supplementary Figure 3. US7 EV and ST6Gal1 OE cells grown in co-culture with OP9 stromal cells without drug treatment. n=4-6 samples per time point. Cell proliferation was measured using CellTiterGlo (Promega). ATP concentrations were calculated based on a standard curve with ATP. Differences are not statistically significant. 2-way ANOVA, Šídák's multiple comparisons test. (**A**) Co-culture with wild type OP9 cells (**B**) co-culture with Galectin-1 knockout OP9 cells.

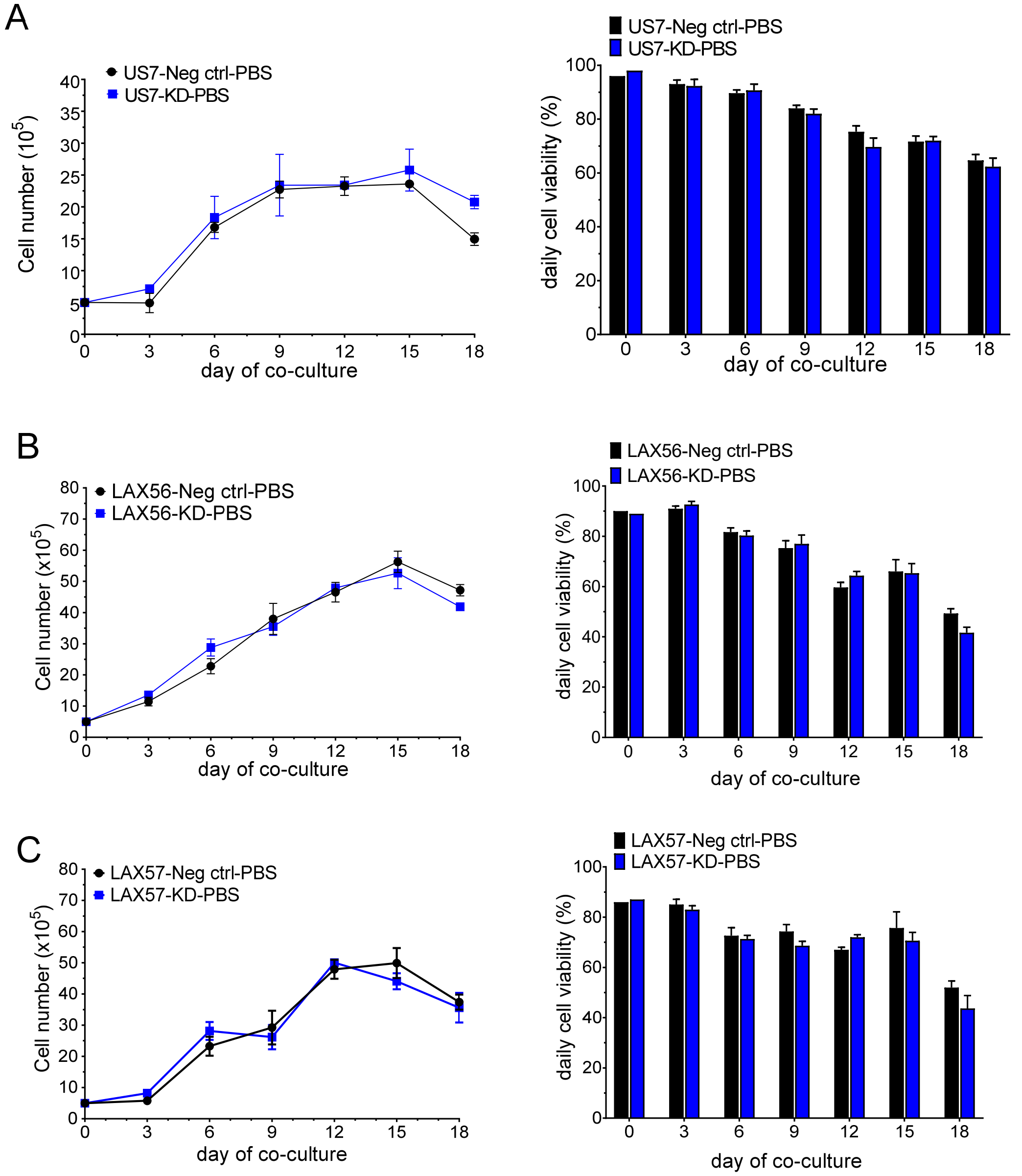

Supplementary Figure 4. Cell counts and viability of US7, LAX56 and LAX57 negative control and ST6Gal1 knockdown BCP-ALL cells grown in co-culture with wt OP9 stromal cells. Cells were counted using Trypan blue exclusion. Viability is defined as the percentage of Trypan-blue excluding cells/total number of cells. Upon extended culture, cell numbers and viability tended to decrease due to overcrowding. (**A**) US7 negative control and ST6Gal1 knockdown cells. (**B**) LAX56 negative control and ST6Gal1 knockdown cells. (**C**) LAX57 negative control and ST6Gal1 knockdown cells. n=3 samples per time point.

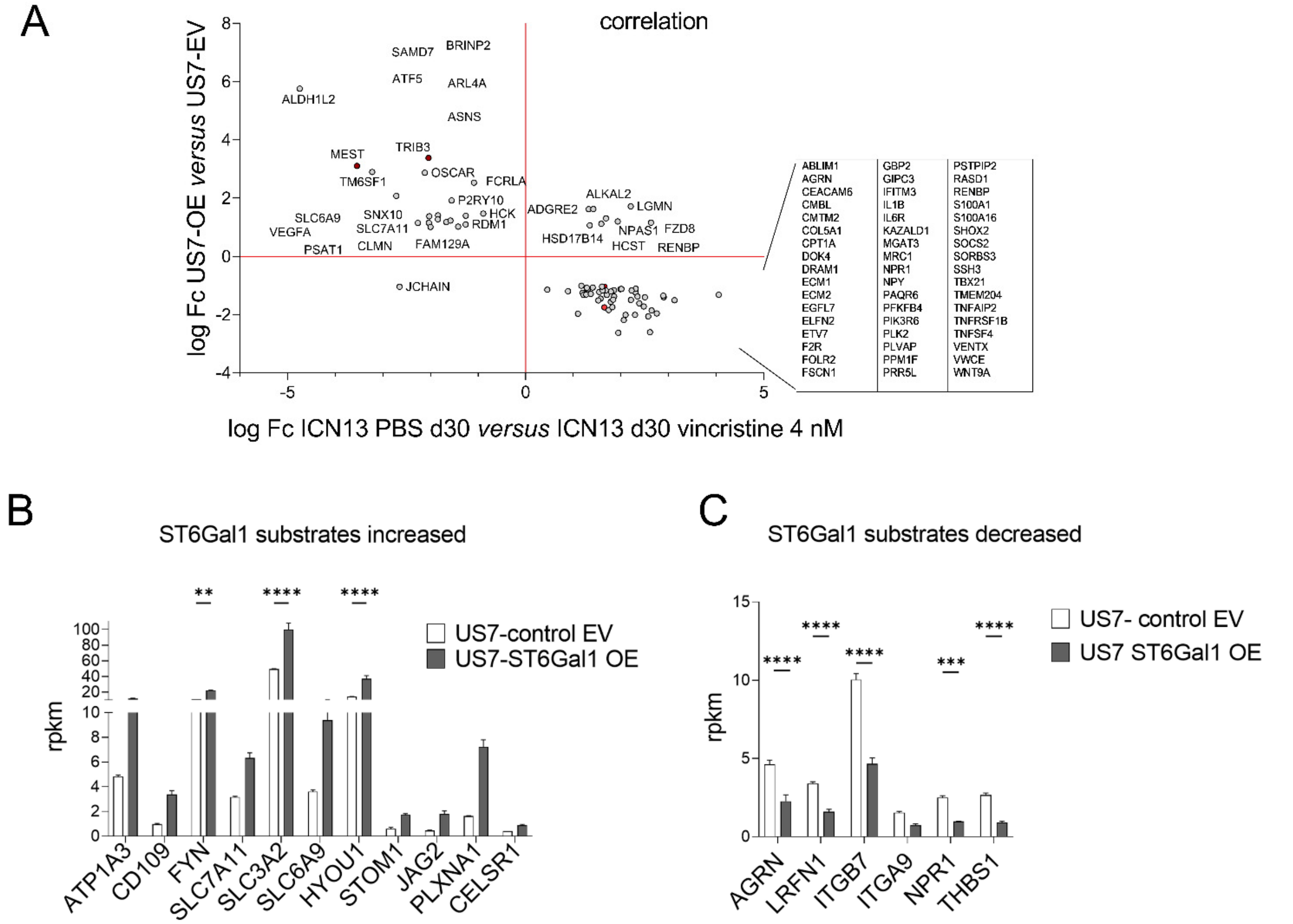

Supplementary Figure 5. Transcriptome comparison of US7 ST6GAL1 OE and EV cells by RNA-seq. (A) Overlap between genes regulated during long-term 30-day resistance development to 4 nM vincristine in ICN13 cells (Oliveira et al, in preparation) and ST6Gal1 overexpression in US7 cells (this study). **(B, C)** Genes encoding glycoproteins that have been identified as substrates of ST6Gal1 in other cell types. Graphs show mean ±SEM of normalized rpkm expression values of the indicated genes. 2-way ANOVA, Sidak's multiple comparison test, adjusted p values. **p<0.01; ***p<0.001; ****p<0.0001.

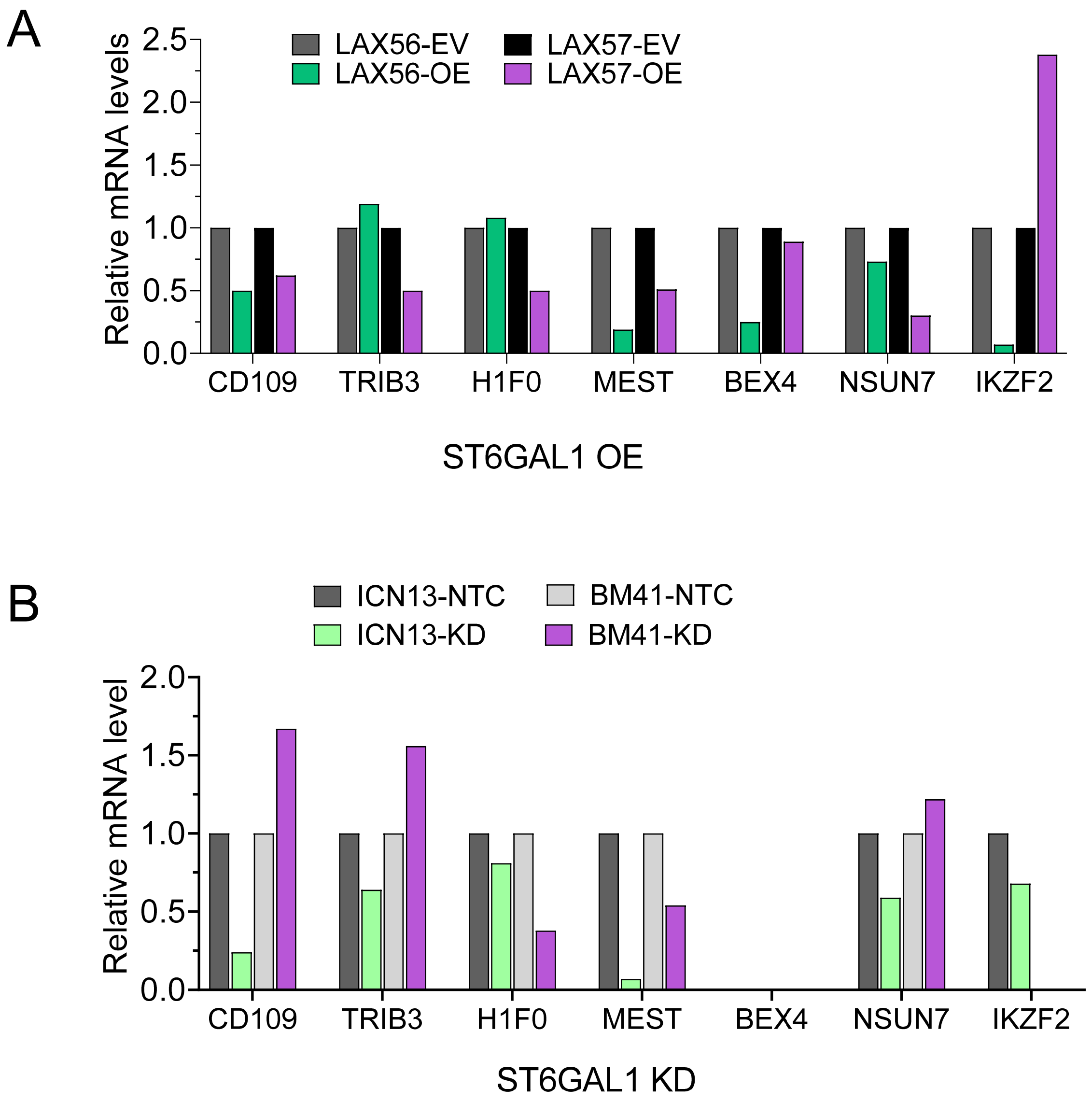

Supplementary Figure 6. Real-time RT-PCR for expression of selected genes that had been also measured in US7 ST6Gal1 OE and KD cells (Figure 8). (**A**) LAX56 and LAX57 cells with ST6Gal1 overexpression (OE) compared to empty vector-transduced cells (EV). (**B**) ICN13 and BM41 ST6Gal1 knockdown (KD) or control negative control (NTC) cells. Values for the EV or NTC samples were set to 1 and results are expressed as fold change. Note: BEX4 is X-linked.

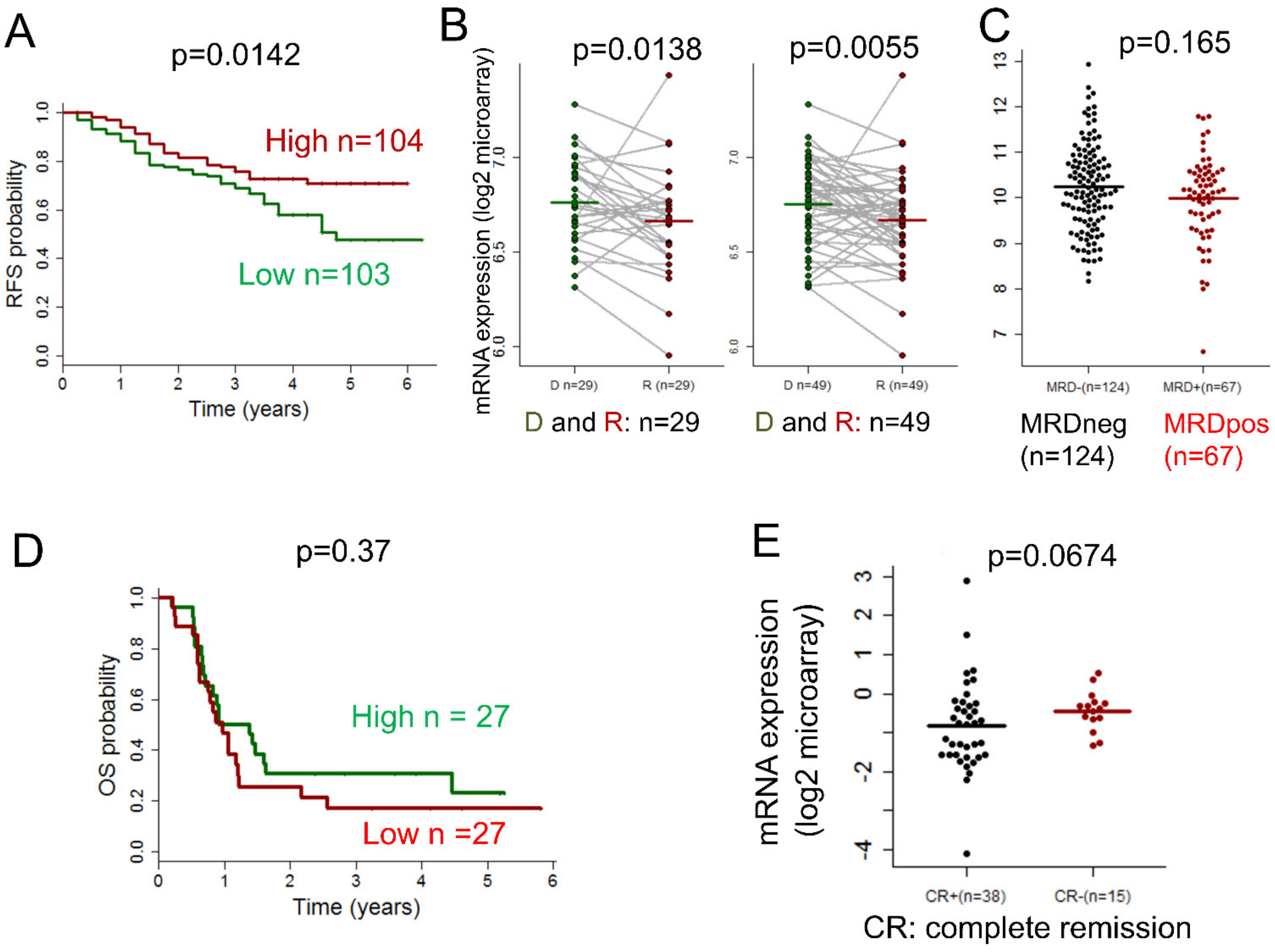

Supplementary Figure 7. Clinical correlates with *ST6GAL1* expression in BCP-ALL samples. (**A**) Childhood ALL COG P9906. n= 207; GSE11877. High *ST6GAL1* expression (probe set 201998_at) correlates with increased relapse-free survival (RFS) probability. p-value, logrank test. p=0.0142 (**B**) Childhood ALL COG P9906; GSE28460. Left, n=29 pairs of early relapse (<36 months) *vs* diagnosis. p=0.0138 for lower *ST6GAL1* in relapse. Right, n=49 pairs of diagnosis *vs* relapse. Average values, p=0.0055 for lower ST6Gal1 in relapse. p-values, paired two-sided Wilcoxon test. (**C**) Childhood ALL COG P9906. n= 207; GSE11877. Minimal residual disease (MRD) negative and positive, probe set 201998_at; p=0.165, ns. (**D** and **E**) Adult ALL ECOG E2993; GSE5314. (**D**) n=54 samples. OS probability is lower for patients with high average *ST6GAL1*. p=0.37, ns. (**E**) Patients who achieved a complete remission (CR+, n=38) had lower *ST6GAL1* expression than those who did not (CR-, n=15). Average values, p = 0.0674, logrank test. **A**, **D**: Median value of *ST6GAL1* expression was determined for all samples. Based on the median value, samples were grouped into those with higher/equal or lower than the median value.

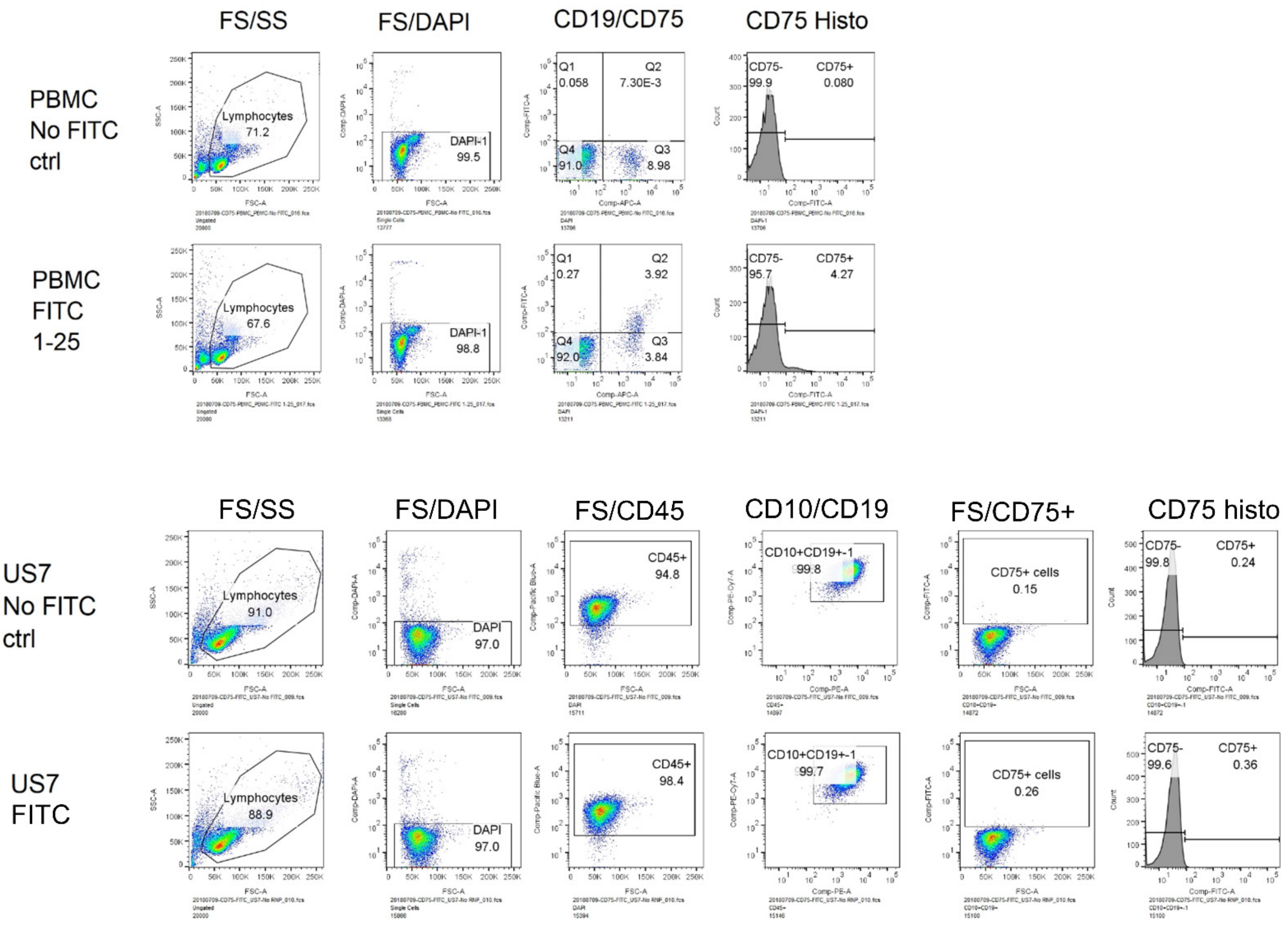

Supplementary Figure 8. Expression of CD75 on normal peripheral blood CD19+ mononuclear cells and US7 BCP-ALL cells. Plots show the gating strategy for each image. CD75 antibody was titrated and a 1:25 dilution was used here. In the normal PBMC population, around 8% of the live cells in the lymphocyte gate are CD19+ (Q2+Q3). Around 50% of the CD19+ cells are also CD75+ (Q2).

Supplementary Table 1. BCP-ALLs used in this study

name	Origin/stage	genetics	Further information
RS4;11	Cell line	MLL-AF4	https://web.expasy.org/cellosaurus/CVCL_0093
US7/LAX7	*PDX-derived, dx	Ph-like	PMID 33531346 Table S3
US7R/LAX7R	*PDX-derived, rel	Ph-like, $KRAS^{G12V}$	PMID 33531346 Table S3
TXL2	*PDX-derived, dx	Ph-positive	PMID 26073130, 25799995
ICN12	*PDX-derived	TCF3-PBX1	PMID 33531346 Table S3
ICN3	*PDX-derived		PMID 32699415 Table S5
ICN13	*PDX-derived	MLL-AF4	PMID 32699415 Table S5
SUP-B15	Cell line	Ph-positive	https://web.expasy.org/cellosaurus/CVCL_0103
Tom1	Cell line	Ph-positive	https://web.expasy.org/cellosaurus/CVCL_1895
Nalm1	Cell line	Ph-positive	https://web.expasy.org/cellosaurus/CVCL_0091
JFK125	*PDX-derived, dx	Ph-like P2RY8-CRLF2	PMID 32699415 Table S5
JFK125R	*PDX-derived, rel	Ph-like P2RY8-CRLF2	PMID 32699415 Table S5
SF06	*PDX-derived, dx	MLL-ENL	PMID 32699415 Table S5
SF06R	*PDX-derived, rel	MLL-ENL	PMID 32699415 Table S5
LAX56	Grown on OP9, rel	t(Y;7)(p11.3;p13)	PMID: 26360058
LAX57	Grown on OP9, dx	t(1;9)(q44;p22)	PMID: 26360058

*passaged in NSG mice, then grown on OP9 stromal cells.
Dx, diagnosis; rel, relapse; PDX, patient-derived xenograft

Supplementary Table 3. Overview of differentially expressed RNAs in US7 ST6Gal1 OE compared to EV cells

comparison	[a]Increased >2 fold, $p<0.05$	[a]decreased >2 fold, $p<0.05$	[a] total	[b] Lysosomal up/down	[c] autophagy up/down	[d] substrates of ST6Gal1 **(Suppl Fig. 5B, C)**	[e] Transcription factors up/down	[f] Glyco-syl trans-ferases up/down	[g] BCP-ALL d30 vincristine treated **(Suppl. Fig. 5A)**
US7 ST6Gal1 OE versus US7 EV	296	188	484	19	11	17	75	11	78

[a] We analyzed 19,862 protein-encoding genes, of which 10,470 were defined as expressed [rpkm>1] in these cells.
[b] The lysosomal genes category includes 828 genes (1)
The stressome genes category includes 457 genes (2)
[c] The autophagy genes category includes 449 genes (3)
[d] 383 glycoproteins were identified as substrates of ST6Gal1 in three different cell types (4-6). Overlap with this data set, also see Suppl. Fig. 5B.
[e] The transcription factor category includes 2764 genes (7)
[f] The glycosyltransferase category includes 221 genes (8).
[g] ICN13 BCP-ALL cells treated for 30 days with vincristine had differential expression of 373 genes with reduced and 575 genes with increased expression compared to d30 PBS samples [Oliveira et al in preparation]. Overlap with this data set, also see Suppl. Fig. 5A.

Literature cited

1. Wyant GA, Abu-Remaileh M, Frenkel EM, Laqtom NN, Dharamdasani V, Lewis CA, et al. NUFIP1 is a ribosome receptor for starvation-induced ribophagy. Science. 2018;360(6390):751-8.
2. Kuechler ER, Budzynska PM, Bernardini JP, Gsponer J, Mayor T. Distinct Features of Stress Granule Proteins Predict Localization in Membraneless Organelles. J Mol Biol. 2020;432(7):2349-68.
3. Li X, Yu W, Qian X, Xia Y, Zheng Y, Lee JH, et al. Nucleus-Translocated ACSS2 Promotes Gene Transcription for Lysosomal Biogenesis and Autophagy. Mol Cell. 2017;66(5):684-97 e9.
4. Yu SH, Zhao P, Sun T, Gao Z, Moremen KW, Boons GJ, et al. Selective Exo-Enzymatic Labeling Detects Increased Cell Surface Sialoglycoprotein Expression upon Megakaryocytic Differentiation. J Biol Chem. 2016;291(8):3982-9.
5. Sun T, Yu SH, Zhao P, Meng L, Moremen KW, Wells L, et al. One-Step Selective Exoenzymatic Labeling (SEEL) Strategy for the Biotinylation and Identification of Glycoproteins of Living Cells. J Am Chem Soc. 2016;138(36):11575-82.
6. Capicciotti CJ, Zong C, Sheikh MO, Sun T, Wells L, Boons GJ. Cell-Surface Glyco-Engineering by Exogenous Enzymatic Transfer Using a Bifunctional CMP-Neu5Ac Derivative. J Am Chem Soc. 2017;139(38):13342-8.
7. Lambert SA, Jolma A, Campitelli LF, Das PK, Yin Y, Albu M, et al. The Human Transcription Factors. Cell. 2018;172(4):650-65.
8. Zhu Y, Groth T, Kelkar A, Zhou Y, Neelamegham S. A GlycoGene CRISPR-Cas9 lentiviral library to study lectin binding and human glycan biosynthesis pathways. Glycobiology. 2021;31(3):173-80.

Supplementary Table 4. Data availability

Description	Source	Accession [URL]
Figure 2A.	Gu et al. PMID 30643249	https://pecan.stjude.cloud/static/hg19/pan-all/Normal.HTSeq.zip
Figure 2B. Expression of ST6GAL1 in different mouse hematopoietic subsets n=3 per sample. Affymetrix gene array probe set ID 10434758	PMID 21307297	http://rstats.immgen.org/Skyline_microarray/skyline.html<br><br>https://www.ncbi.nlm.nih.gov/geo/query/acc.cgi?acc=GSE15907
Figure 2C.	Gu et al. PMID 30643249	https://pecan.stjude.cloud/static/hg19/pan-all/B-ALL-subtyping.FPKM-UQ10-log2.txt.zip
Figure 2D.	Yeoh et al PMID 29808917	https://www.ncbi.nlm.nih.gov/geo/query/acc.cgi?acc=GSE67684
Figure 6. RNA-seq of US7-EV and US7-ST6Gal1 OE cells	This study	https://www.ncbi.nlm.nih.gov/geo/query/acc.cgi?acc=GSE185611
